# Spatial mapping of gene expression in the salivary glands of the dengue vector mosquito, *aedes aegypti*

**DOI:** 10.1186/1756-3305-4-1

**Published:** 2011-01-04

**Authors:** Jennifer Juhn, Unsar Naeem-Ullah, Bruno Augusto Maciel Guedes, Asif Majid, Judy Coleman, Paulo Filemon Paolucci Pimenta, Waseem Akram, Anthony Amade James, Osvaldo Marinotti

**Affiliations:** 1 Department of Molecular Biology and Biochemistry, University of California, Irvine, USA; 2 Department of Agricultural Entomology, University of Agriculture, Faisalabad, Pakistan; 3 Laboratory of Medical Entomology, Centro de Pesquisas Rene Rachou, Fundação Oswaldo Cruz, MG, Brazil; 4 Department of Morphology, Universidade Federal de Minas Gerais, Belo Horizonte, MG, Brazil; 5 Department of Microbiology and Molecular Genetics, University of California, Irvine, USA

## Abstract

**Background:**

*Aedes aegypti *mosquitoes are the main vectors of dengue viruses to humans. Understanding their biology and interactions with the pathogen are prerequisites for development of dengue transmission control strategies. Mosquito salivary glands are organs involved directly in pathogen transmission to vertebrate hosts. Information on the spatial distribution of gene expression in these organs is expected to assist in the development of novel disease control strategies, including those that entail the release of transgenic mosquitoes with impaired vector competence.

**Results:**

We report here the hybridization *in situ *patterns of 30 transcripts expressed in the salivary glands of adult *Ae. aegypti *females. Distinct spatial accumulation patterns were identified. The products of twelve genes are localized exclusively in the proximal-lateral lobes. Among these, three accumulate preferentially in the most anterior portion of the proximal-lateral lobe. This pattern revealed a salivary gland cell type previously undescribed in *Ae. aegypti*, which was validated by transmission electron microscopy. Five distinct gene products accumulate in the distal-lateral lobes and another five localize in the medial lobe. Seven transcripts are found in the distal-lateral and medial lobes. The transcriptional product of one gene accumulates in proximal- and distal-lateral lobes. Seven genes analyzed by quantitative PCR are expressed constitutively. The most abundant salivary gland transcripts are those localized within the proximal-lateral lobes, while previous work has shown that the distal-lateral lobes are the most active in protein synthesis. This incongruity suggests a role for translational regulation in mosquito saliva production.

**Conclusions:**

Transgenic mosquitoes with reduced vector competence have been proposed as tools for the control of dengue virus transmission. Expression of anti-dengue effector molecules in the distal-lateral lobes of *Ae. aegypti *salivary glands has been shown to reduce prevalence and mean intensities of viral infection. We anticipate greater efficiency of viral suppression if effector genes are expressed in all lobes of the salivary glands. Based on our data, a minimum of two promoters is necessary to drive the expression of one or more anti-dengue genes in all cells of the female salivary glands.

## Background

Mosquito (Diptera, Culicidae) salivary glands have been studied extensively for their roles in blood feeding and pathogen transmission to vertebrate hosts. A number of morphological [1-6] and biochemical studies [7-12] describe salivary gland structure and molecular composition. In addition, transcriptomes and proteomes have been described for many mosquito species, including the dengue vector, *Aedes aegypti *[[13-15], http://exon.niaid.nih.gov/transcriptome.html].

The salivary glands of adult mosquitoes are sexually dimorphic and it is clear that their structural and functional differences enable females to engage successfully in hematophagy [16,17]. The salivary glands of adult female *Ae. aegypti *have a distinctive tri-lobed structure consisting of a single medial and two lateral lobes. Each lobe comprises a secretory epithelium surrounding a salivary duct into which saliva is released.

The complex mosquito saliva is produced by secretory cells of the proximal and distal regions of the lateral lobes and the distal region of the medial lobe. The secretory products collect in extracellular secretory cavities that are connected by openings to the salivary duct. The mosquito salivary glands produce and secrete molecules with diverse enzymatic, anti-hemostatic and anti-inflammatory activities, which help in the acquisition of blood meals from vertebrate hosts, as well as for the digestion of sugar and nectar meals [15,18]. Additionally, mosquito saliva modulates vertebrate immune responses potentially increasing virus transmission, host susceptibility, viremia, disease progression and mortality [19-21].

Despite the extensive knowledge acquired thus far about mosquito saliva components and their functions, little is known about the spatial-specificity of expression of the corresponding genes in the salivary glands. Here we report the hybridization *in situ *patterns of 30 genes expressed in the salivary glands of adult *Ae. aegypti *females, the identification of a new cell type located in the proximal portion of the lateral lobes, and discuss the application of such knowledge for enhancing efforts to interfere with dengue virus transmission.

## Materials and methods

### Mosquitoes

The Liverpool strain of *Ae. aegypti *(L.) was used for all gene amplification and hybridization *in situ *experiments and the PPCampos strain was used in the transmission electron microscopy experiments. Standard rearing procedures were used [22]. Briefly, mosquitoes were reared at 28°C, 80% humidity with 18 h light, 6 h dark. Raisins were provided as a sugar source and females were fed on anesthetized mice.

### Tissue dissection and RNA isolation

Salivary glands were dissected from adult females in phosphate-buffered saline (PBS), frozen in Trizol reagent (Invitrogen, Carlsbad, CA) and stored at -80°C prior to RNA extraction. Total RNA was extracted, dissolved in RNAse-free H_2_O and treated for 30 min at 37°C with RQ1 DNAse (Promega, Madison, WI).

### cDNA cloning and RNA probe synthesis

The One-step RT-PCR kit (Qiagen, Valencia, CA) was used for cDNA amplification reactions. Primer pairs were designed using the Primer3 Plus primer design software http://www.bioinformatics.nl/cgi-bin/primer3plus/primer3plus.cgi to amplify products of a minimum of 500 nucleotides whenever possible. Sequences of all oligonucleotide primers are listed in Additional file 1. The reaction mixtures were incubated at 50°C for 30 min and 15 min at 95°C. Amplification conditions were 3 min at 95°C followed by 30 cycles of 30 s at 95°C, 30 s at 60°C and 1 min at 72°C. RT-PCR products were cloned into the pCR^®^4-TOPO^® ^cloning vector (Invitrogen) and sequenced to confirm their identity. Digoxygenin (DIG)-labeled antisense RNA probes for salivary gland gene products were synthesized *in vitro *using T3 or T7 RNA polymerases (Ambion, Austin, TX).

### Whole-mount salivary gland preparation and hybridization in situ

Salivary glands were dissected in PBS from adult female mosquitoes 4 days post emergence. Tissues were fixed with 4% formaldehyde in PBS immediately after dissection. Post-fixation treatment, hybridization and signal detection were conducted as described previously [23], with the exception that Proteinase K treatment was omitted.

### Electron microscopy

Salivary glands of 3-5 day old female mosquitoes were dissected and fixed for 2 hours in 2.5% glutaraldehyde in 0.1 M cacodylate buffer, pH 7.2. Samples were post-fixed in 1% osmium tetroxide in 0.8% potassium ferricyanide and 0.1 M cacodylate buffer, pH 7.2, dehydrated in a series of acetone (30 to 100%) and embedded in EPON-812 resin (Electron Microscopy Sciences, Hatfield, PA). Thin sections (60-70 nm) were stained with uranyl acetate and lead citrate, and were examined in a Jeol^® ^JEM-1011 electron microscope (Jeol, Tokyo, Japan).

Alternatively, salivary glands were fixed in glutaraldehyde as described above, rinsed in PBS, dehydrated in graded ethanol (30 to 100%) and immersed for 48 hours in 2.0% phosphotungstic acid (PTA) in absolute ethanol. Glands were washed subsequently in ethanol (2X for 10 min), ethanol and acetone (1:1) for 10 min and absolute acetone (2X for 10 min). After dehydration the material was embedded in EPON-812 resin (Electron Microscopy Sciences), and thin sections (60-70 nm) were examined in a Jeol^® ^JEM-1011 electron microscope.

### Real-time quantitative RT-PCR

Salivary glands were dissected from adult female mosquitoes at various time points: 1 day and 4 days post emergence (PE), and 6 h, 24 h, and 48 h post blood feeding. Mosquitoes were blood-fed on anesthetized mice for 30 min, 4-5 days PE. Three biological replicates, each consisting of salivary glands from 20 mosquitoes, were collected for each time point. cDNA was synthesized from DNAse-treated total RNA from pooled samples of 20 salivary gland pairs using SuperScript III (Invitrogen) and oligo dT. All primers were designed to flank the start of the 3'-end untranslated region to increase target specificity. Oligonucleotide primers were designed to amplify a 97 nucleotide fragment of AAEL003396, a constitutively-expressed reference gene encoding the 60 S ribosomal protein L32 (*rpL32*). Quantitative PCR was performed with an iQ5 real-time PCR detection system (BioRad) with iQ-SyberGreen Super Mix (BioRad). The relative 2^-ΔΔCt ^method [24] was used to determine fold-changes of transcript abundance in salivary glands at various time points compared to the calibrator time point 1 d PE. Mean fold-changes and standard deviations from three biological replicates were calculated, except time point 4 d PE for AAEL009670, AAEL000726 and AAEL006347, where values were calculated from two biological replicates. A one-way ANOVA was used to study association between the experimental time course and level of transcript accumulation. Pair-wise comparisons with the calibrator 1 d PE were performed using Dunnett's test. Analysis was done using JMP Statistical Discovery Software (JMP, Version 8. SAS Institute Inc., Carey, NC). A *P *value of ≤0.05 was considered to be significant.

## Results

### Five distinct groups of spatially-restricted transcript accumulation are identified in the salivary glands of Ae. aegypti

**Figure 1:**
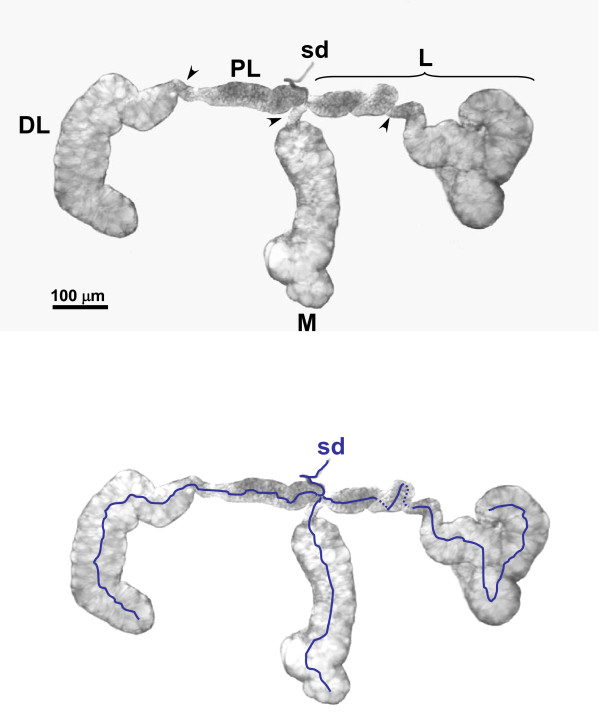
**Whole-mount of a single salivary gland dissected from a female *Aedes aegypti *mosquito**. The salivary gland is comprised of two lateral lobes and a single, centrally-located medial lobe, **M **. The lateral lobes, **L **are further defined into two regions: proximal-lateral, **PL **and distal-lateral, **DL**. Intermediate, or neck regions are indicated with arrowheads (top panel). The salivary duct (**sd) **that connects all ducts of the salivary gland lobes is illustrated (bottom panel).

**Figure 2:**
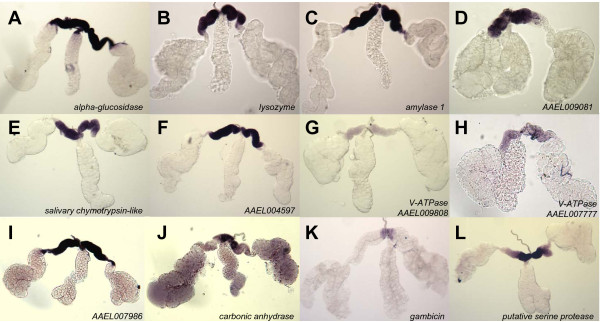
**Hybridizations *in situ *for twelve genes expressed in the proximal-lateral lobes of whole-mount *Ae. aegypti *salivary glands**. **A**. *alpha-glucosidase *(AAEL000392); **B**. lysozyme (AAEL009670); **C**. *amylase 1 *(AAEL006719); **D**. AAEL009081; **E**. salivary chymotrypsin-like (AAEL015294); **F**. AAEL004597; **G**. V-ATPase (AAEL009808); **H**. V-ATPase (AAEL007777); **I**. AAEL007986; **J**. *carbonic anhydrase *(AAEL010893); **K**. *gambicin *(AAEL004522); **L**. putative serine protease (AAEL005596).

An initial list of candidate genes was compiled from a catalogue of salivary gland transcripts found to be expressed at high levels within the glands of *Ae. aegypti *[14] and 30 were selected for hybridization *in situ *analyses. Hybridizations of digoxigenin-labeled anti-sense RNA to whole-mount salivary glands dissected from adult female mosquitoes showed that transcripts accumulate in specific lobes of the salivary gland. The paired salivary glands of female *Ae. aegypti *are composed of morphologically-distinct lobes, two lateral lobes consisting of proximal, intermediate and distal regions, and a medial lobe consisting of a neck and distal region (Figure 1). Distinct mRNA spatial localization patterns can be classified into five groups: proximal-lateral lobe, distal-lateral lobe, all-lateral lobe, medial lobe, and those that accumulate in both the distal-lateral and medial lobes (distal-lateral/medial lobe). The proximal-lateral group comprises 12 of the 30 genes examined (Figure 2): *alpha-glucosidase *(AAEL000392); a lysozyme (AAEL009670); amylase *1 *(AAEL006719); a salivary chymotrypsin-like gene (AAEL015294); two putative vacuolar-type H^+^-ATPase subunits (AAEL009808 and AAEL007777); three genes with unknown functions (AAEL009081, AAEL004597 and AAEL007986); *carbonic anhydrase *(AAEL010893); *gambicin *(AAEL004522); and a putative serine protease (AAEL005596). The last three genes belong to a sub-class of the proximal-lateral lobe group that is transcribed within the anterior-most portion of the proximal-lateral lobes (Figure 2J-L). The variation in the intensity of the signals among the samples in all of the images displayed does not represent quantitative differences in transcription product abundance.

**Figure 3:**
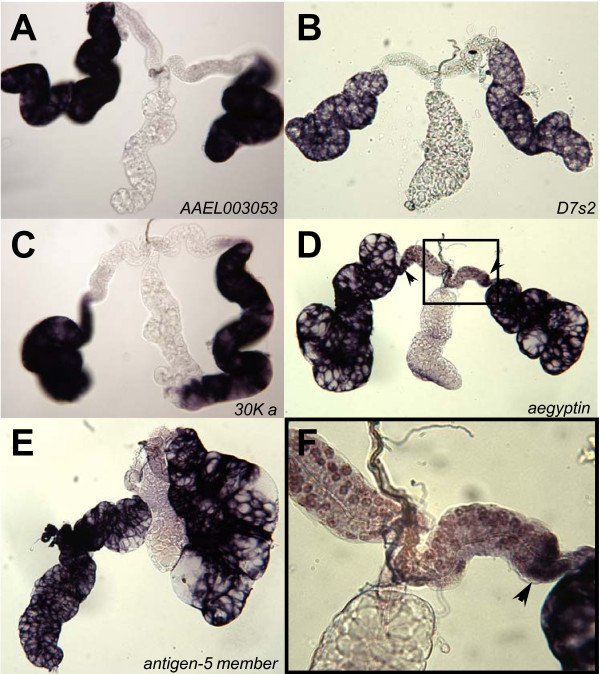
**Hybridizations *in situ *for five genes expressed in the distal-lateral lobes of whole-mount *Ae. aegypti *salivary glands**. **A**. putative orthologue of *Culex quinquefasciatus AG5-3 *(AAEL003053); **B**. *D7s2 *(AAEL006423); **C**. a putative 30 kDa allergen-like protein *30K a *(AAEL010228); **D**. *aegyptin *(AAEL0010235), hybridization signal present in restricted regions outside of the distal lateral lobes (black arrows); **E**. *antigen 5 member *(AAEL000793); **F**. 4X enlargement (from 2D) of hybridization signal for *aegyptin *mRNA in the distal tip of the proximal-lateral lobe and intermediate region of the lateral lobe.

The distal-lateral lobe group includes five of the 30 genes (Figure 3): a member of the *D7 *family, *D7s2 *(AAEL006423); putative 30 kDa allergen-like proteins, *30K a *(AAEL010228) and *aegyptin *(AAEL010235), which also has been designated *30K b *[25]; an antigen-5 member (AAEL000793); and AAEL003053, a putative orthologue of a predicted salivary secreted antigen-5 precursor (AG5-3) from *Culex quinquefasciatus*. The mRNAs of these genes accumulate only in the cells of the distal-lateral lobes, except for the transcripts of *aegyptin*, which also accumulate in the intermediate region and distal tip of the proximal-lateral lobes (Figure 3D and 3F). The corona-like digoxigenin-staining patterns observed in the distal region of the salivary gland (for example Figure 3B, D and 3E) reflects hybridization to mRNA in the cytoplasm of the secretory cells. Staining indicating hybridization to mRNA is not expected within the extracellular salivary cavities located apically to the secretory cells of the salivary glands.

**Figure 4:**
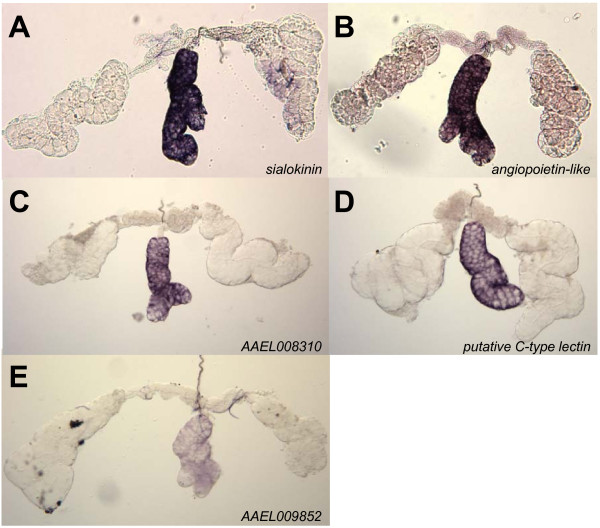
**Hybridizations *in situ *for five genes expressed in the medial lobes of whole-mount *Ae. aegypti *salivary glands**. **A**. *sialokinin *(AAEL000229); **B**. *angiopoietin-like *(AAEL00726); **C**. AAEL008310; **D**. a putative C-type lectin (AAEL000533); **E**. AAEL009852.

There are five members of the medial lobe group: *sialokinin*, a salivary vasodilatory protein (AAEL000229); a gene encoding a predicted protein with angiopoietin-like features (AAEL000726); a putative C-type lectin (AAEL000533) and two genes with unknown functions (AAEL008310 and AAEL009852) (Figure 4).

**Figure 5:**
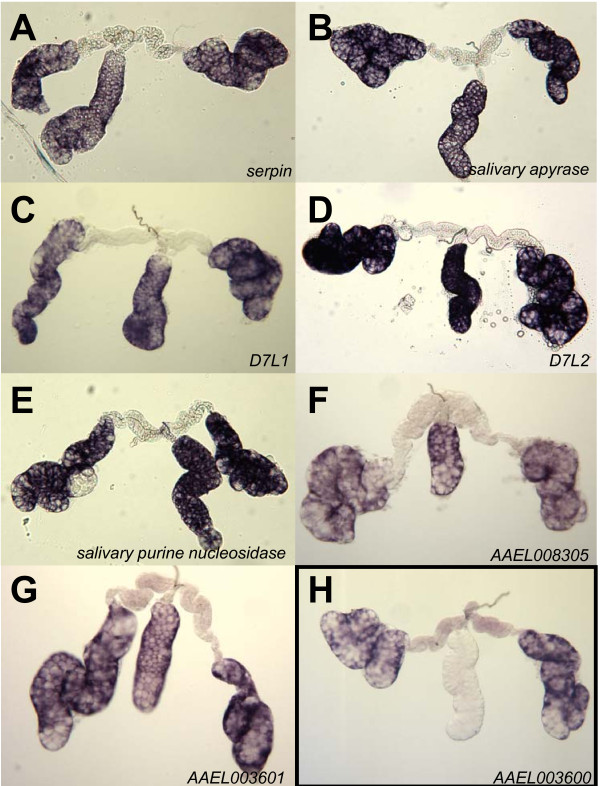
**Hybridizations *in situ *for seven genes expressed in the distal-lateral and medial lobes (A-G) and one gene expressed in proximal and distal-lateral lobes (H) of whole-mount *Ae. aegypti *salivary glands**. **A**. *serpin *(AAEL003182); **B**. *salivary apyrase *(AAEL006347); **C**. *D7L1 *(AAEL006417); **D**. *D7L2 *(AAEL006424); **E**. *salivary purine nucleosidase *(AAEL006485); **F**. AAEL008305; **G**. AAEL003601; **H**. AAEL003600.

The distal-lateral/medial group comprises a *serpin *(AAEL003182), *salivary apyrase *(AAEL006347), *D7L1 *(AAEL006417), *D7L2 *(AAEL006424), a salivary purine nucleosidase (AAEL006485) and two genes with unknown functions (AAEL083050 and AAEL003601) (Figure 5A-G). The last group consists of a single transcript of unknown function (AAEL003600-RA) that accumulates in both proximal and distal-lateral lobes (Figure 5H).

### Morphological characterization of mosquito salivary gland cells

The spatial patterns of transcript accumulation determined in this study support previously-described, functionally-distinct regions of the mosquito salivary glands [1,3,10,16]. In addition, a previously-undescribed region has a distinct hybridization pattern comprising the most anterior portion of the proximal-lateral lobes (Figure 2J-L). Electron microscopy techniques confirm the presence of a morphologically-distinct cell type in the region.

**Figure 6:**
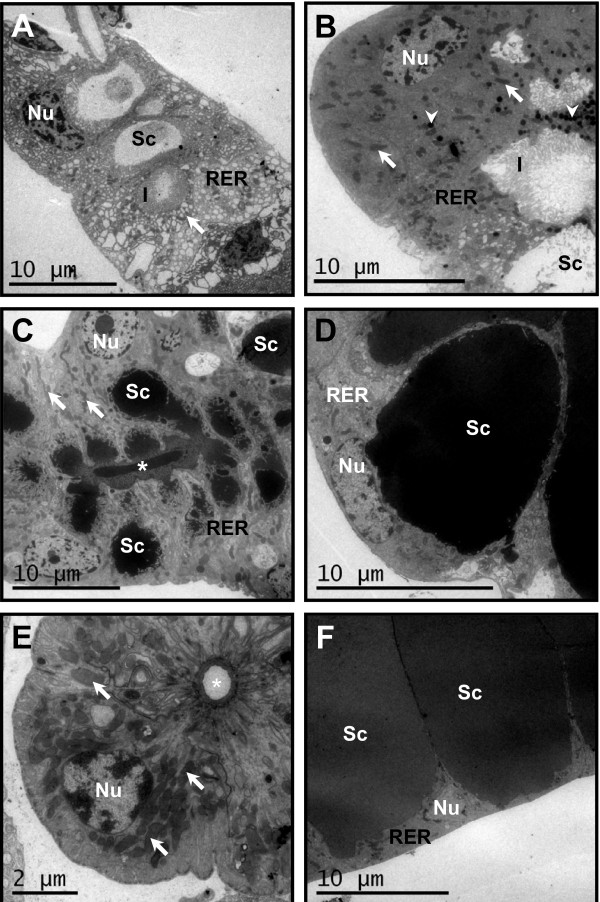
**Electron microscopy of cross-sections of dissected *Aedes aegypti *salivary glands**. **A**. Electron micrograph using cytochemical techniques. Anterior portion of the proximal lateral lobe showing a cell with the nucleus (**Nu**) located basally in the cytoplasm, a developed rough endoplasmic reticulum (**RER**) and finely granular secretory material. Additional features indicated are: secretory cavity (**Sc**); mitochondria (arrow) and invaginations (**I**). **B-F**. Micrographs produced using conventional electron microscopy. **B**. Middle portion of the proximal lateral lobe showing electron-dense cells, **Nu **in the basal region of the cell and a large amount of mitochondria (arrows) and secretory vesicles (arrowheads). **C**. The distal portion of the lateral lobe showing cells with **Nu **and little condensed chromatin, prominent nucleoli and elongated and rounded mitochondria (arrows). The **Sc **has many invaginations of the membrane with fine and electron-dense secretions. The salivary duct also is indicated (asterisk). **D**. Electron micrograph of cells from the distal-lateral lobe. The cells contain a concentration of **RER **and large **Nu **with prominent nucleoli. **Sc **contain dark homogeneous secretions and membrane invaginations. **E**. Proximal portion of the medial lobe showing large and central **Nu **and the presence of many mitochondria (arrows) associated with basal membrane invaginations. The salivary duct also is indicated (asterisk). **F**. Electron micrograph of cells from the distal region of the medial lobe. The **Nu **has little condensed chromatin and the cytoplasm has dispersed **RER **cisternae with rectilinear and irregular organization. The **Sc **have irregular borders.

Transmission electron microscopy of PTA-stained sections showed two distinguishable cell types in the proximal regions of the lateral lobes (Figure 6A). The cells found in the anterior-most portion of the proximal lateral lobes exhibit nuclei with a large mass of condensed chromatin. In these cells, few mitochondria were found between the cisterns of RER. The extracellular secretory cavities of these cells contain a fine granular material and a large number of membranous invaginations. The other cells found in the proximal lateral lobes (Figure 6B) have nuclei displaying little condensed chromatin, cytoplasm rich in mitochondria with prominent and electron-dense vesicles, mainly located near the secretory cavities. In contrast to the first cell type, the RER in these cells is well developed. The secretory cavities of these cells display numerous large invaginations of the membrane and the cavity lumen was found to contain fine granular material as seen in the other cell type.

The distal regions of the lateral and medial lobes display an overall architecture consistent with previous morphological descriptions of the organ, with cells surrounding secretory cavities containing uniform and electron-dense secretory material (Figure 6C, D and 6F). The cells of the proximal region of the medial lobe contain a high number of mitochondria that can be enclosed by deep folds of membrane extending to the reticulate basal cell membrane (Figure 6E).

### Quantitative real-time RT-PCR indicates constitutive gene expression for seven salivary gland genes

**Figure 7:**
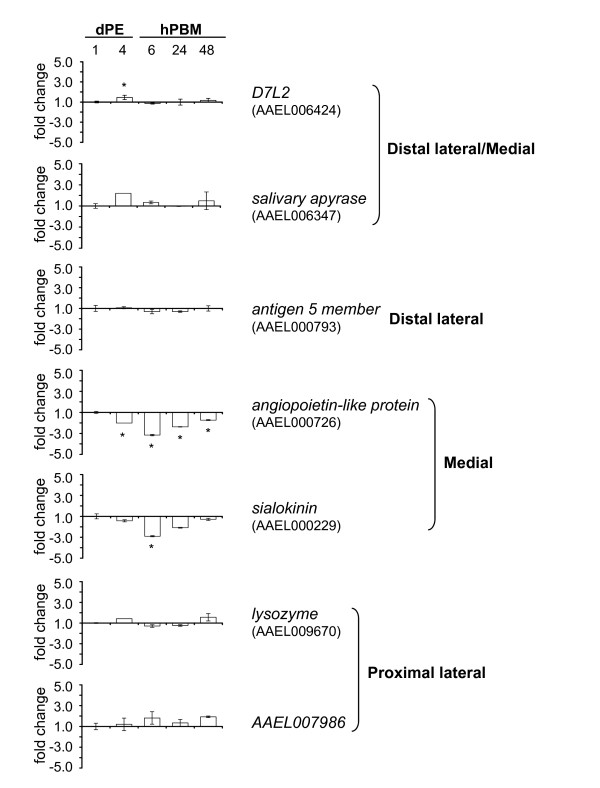
**Quantitative real time RT-PCR analysis of seven salivary gland genes of various spatial expression pattern groups**. Mean fold-changes in salivary gland gene expression are indicated for various time points: one and four days post emergence, **dPE **and 6, 24, and 48 hours post blood meal, **hPBM**. All fold-changes are relative to the endogenous reference gene *rpL32 *(AAEL003396) and all time points are compared to the calibrator time point 1 dPE. Standard deviations from three biological replicates are expressed with error bars, except time point 4 dPE for AAEL006347, AAEL000726 and AAEL009670, where values were calculated from two biological replicates. ANOVA analysis and pair-wise Dunnett's tests were performed. Statistically-significant differences having a *P *value of ≤0.05 are indicated (asterisk).

Standard quantitative real-time RT-PCR procedures were performed and a comparative method with the reference gene *rpL32 *was used to determine relative fold changes in transcript accumulation for genes of four spatial groups of salivary gland-expressed genes. Genes selected for analysis include two expressed in proximal-lateral lobes, a lysozyme gene (AAEL009670) and a gene encoding a putative 18.5 kDa secreted protein (AAEL007986); one gene from the distal-lateral group, antigen-5 member (AAEL000793); two from the medial group, a gene encoding an angiopoietin-like protein (AAEL000726) and sialokinin (AAEL000229); and two genes from the distal lateral and medial group, D7L2 (AAEL006424) and salivary apyrase (AAEL006347). All genes except for *salivary apyrase *were expressed at levels greater than *rpL32 *and for the majority of genes analyzed, fold-changes in gene expression did not vary significantly over the experimental time course. However, the two medial group genes, angiopoietin-like and sialokinin, did show significantly (p ≤ 0.05) lower accumulation levels following blood feeding based on ANOVA analysis and Dunnett's test with pair-wise comparisons using 1 dPE as the calibrator (Figure 7).

## Discussion

### Mosquito salivary glands

Transcript localization patterns within *Ae. aegypti *salivary glands had been determined previously for only five genes expressed abundantly and whose functions have been characterized using molecular and biochemical techniques [26-30]. Here we report the results of a systematic hybridization *in situ *approach to group genes based on the localization patterns of their transcripts. Our findings show the spatial distribution of 30 genes that have localization patterns unique to one or more lobes of the salivary gland as well as novel patterns for distinct sub-glandular regions. The hybridization *in situ *signals observed fall into five spatial pattern groups: proximal-lateral (twelve genes), distal-lateral (five genes), medial (five genes), distal-lateral/medial (seven genes), all lateral (one gene), and one subclass of the proximal-lateral group in which gene products accumulate in the anterior-most portion of the proximal-lateral lobe (three genes). This diversity of spatially-restricted expression patterns reveals a more elaborate picture of salivary gland-specific gene regulation and an alternative to the view of a simplified tri-regional compartmentalization of the salivary gland [31]. Moreover, our findings support the argument for a need to better understand gene functions in conjunction with spatial distribution.

Constituents of female mosquito saliva have various functions related to their secretion during sugar- or blood-feeding, and from previous observations it has been postulated that these salivary constituents are compartmentalized in their expression to particular lobes of the salivary glands. Genes shown previously by both enzymatic and hybridization *in situ *analyses to be expressed in the proximal-lateral lobes of *Ae. aegypti *salivary glands, such as *1,4-alpha-glucosidase *(*maltase I) *and *amylase 1*, are related to the digestion of the sugar meal [27,28,32,33]. These patterns of expression are corroborated by the hybridization *in situ *results presented herein.

Lysozyme is another abundantly-expressed gene product in the proximal regions of the lateral lobes that was identified previously by biochemical analysis of female *Ae. aegypti *salivary glands [34]. It was proposed that this major salivary component acts as a bacteriolytic factor that protects mosquitoes from pathogenic bacteria in the sugar meal during ingestion and storage in the crop. AAEL009670 has been identified as the most abundant lysozyme transcript in the salivary glands of *Ae. aegypti *[14]. *Aedes albopictus*, a near relative of *Ae. aegypti*, also expresses a salivary lysozyme [35]. This corresponding gene is similar (80% identical in nucleotide sequence) to the lysozyme, AAEL009670, described here by hybridization *in situ *and real-time RT-PCR. We propose based on amino acid similarity and tissue localization that these genes are orthologous and correspond to the activity detected by Rossignol and Lueders [34].

Three transcripts, encoding *gambicin*, *carbonic anhydrase *and a putative serine protease accumulate in a novel functional region within the anterior-most portion of the proximal-lateral lobes. Transmission electron microscopy shows that this region contains a cell type that is distinct morphologically from cells in other regions of the salivary gland. Previous work also describes two well-defined cellular types in the most proximal portion of the salivary glands of *Anopheles darlingi *[5]. These findings indicate that a distinct group of differentiated cells in the anterior-most portion of the proximal lateral lobes are present in the salivary glands of both anopheline and culicine mosquitoes. The localization in the anterior-most portion of the proximal-lateral lobes of the transcript for *gambicin*, an anti-microbial gene product involved in mosquito innate immune response, supports the hypothesis that this region of the glands may produce other salivary components that prevent microbial infection during sugar-feeding.

The localization patterns described for *alpha-glucosidase*, *amylase 1*, *lysozyme*, and *gambicin *support the conclusion that genes expressed in the proximal-lateral lobes correspond to sugar-feeding and nectar-related digestive and bacteriocidal functions. However, serine protease-like gene products (AAEL015294 and AAEL005596) containing secretory signal peptides also are expressed in the proximal-lateral lobes. While the majority of protein ingested by female mosquitoes is obtained during blood-feeding it is possible that gene products are needed to metabolize low abundance proteins ingested during sugar feeding. Alternatively, these and other products in the proximal-lateral lobes could be involved in blood feeding, for example enzymes could interact with vertebrate host hemostatic factors. The leech *Haementeria ghilianii *produces a proteolytic enzyme, Hementin, in its salivary glands, which has an anticoagulant activity [36]. The mechanism of action for Hementin involves the cleavage of peptide bonds in fibrinogen. Uncharacterized mosquito salivary enzymes could perform a similar function or play other roles in blood feeding.

In contrast to the proximal regions, the distal regions of the lateral and medial lobes have been shown previously to produce salivary products such as platelet aggregation inhibitors, anticoagulants and vasodilatory agents involved in hematophagy [16,30,37,38]. Hybridization *in situ *confirms the role of the distal-lateral and medial lobes in the expression of genes involved with blood-feeding. Genes such as *salivary apyrase *(AAEL006347), *salivary purine nucleosidase *(AAEL006485), *serpin *(AAEL003182), an antigen-5 family member (AAEL000793), *aegyptin *(AAEL010235), 30-kDa allergen (AAEL0010228), and the D7 family genes all have been shown previously to be major components of hematophagous mosquito saliva and play roles in suppressing human host wound responses, preventing hemostasis and causing host hypersensitivity responses [11,14,29,35,37-42]. The hybridization *in situ *patterns for three members of the D7 gene family show that the mRNA of one short isoform D7s2 (AAEL006423) is localized only in the distal-lateral lobes, while transcripts of two long isoforms, D7L1 (AAEL006417) and D7L2 (AAEL006424), accumulate in distal-lateral and medial lobes. The D7 gene family encodes proteins that bind biogenic amines including histamine, serotonin and norepinephrine and thereby inhibit vasoconstriction and platelet aggregation, while promoting blood-feeding [41].

Two of five genes, sialokinin (AAEL000229) and *angiopoietin-like *(AAEL000726), restricted spatially to only the medial lobe, have described functional domains. *Sialokinin *is a potent vasodilatory tachykinin that has been shown to help maintain blood flow during hematophagy and increase the likelihood of venipuncture during host probing by enlarging target venules and arterioles [30,43,44]. The *angiopoietin-like *gene product investigated here was identified through proteomics approaches and is one of a family of angiopoietin-like variants that are involved in immunity-related responses [14,45]. While all other genes tested by quantitative PCR analysis showed consistent levels of transcription product accumulation, both *sialokinin *and the *angiopoietin-like *medial-lobe gene products were decreased following a blood meal. The reasons for this observation are as of yet unknown.

The intermediate regions of the lateral lobes are not thought to be associated with saliva production [1,3]. The membrane structures of these cells support the interpretation that they actively transport water and other molecules required for the function of the salivary glands. We hypothesized that vacuolar-type H+ -ATPases (V-ATPases) were expressed in the intermediate regions to facilitate salivation. However, hybridization *in situ *analyses showed that transcripts of two different V-ATPase subunits (AAEL007777 and AAEL009808) accumulate in the proximal-lateral lobes, not in the intermediate regions. The presence in the proximal-lateral lobe of these molecules, which serve a basic tissue function for solute transport, support the hypothesis that the proximal regions of the lateral lobes are most similar to the tubular salivary glands of non-hematophagous mosquitoes [46,47]. Moreover, the molecular and physiological mechanisms for proximal-lateral lobe secretions, which are primarily for sugar feeding, may be independent of those related to blood feeding, which are synthesized and accumulate in the distal portions of the lateral and medial lobes [48].

Another surprising finding from our studies is that transcripts identified previously as the most abundant in *Ae. aegypti *salivary glands [14] are localized within the proximal-lateral lobes, not the distal-lateral and medial lobes. Previous work to quantify levels of protein synthesis within the various lobes of the mosquito salivary gland by ^35^S -methionine labeling showed that the distal regions of the lateral lobes followed by the medial lobe were the most translationally active, while the proximal regions of the lateral lobe were much less active [10]. The difference between our findings, which are based on transcript detection, and previous work based on protein detection, suggests a role for translational regulation in modulating mosquito saliva composition.

### Dengue and transgenic mosquitoes

Dengue is the most prevalent mosquito-borne viral disease of humans worldwide, with 3 billion people at risk of infection and ~50 million annual cases of dengue fever [49]. Changing epidemiology continues to challenge the surveillance and prevention of epidemic dengue transmission in Southeast Asia [50,51] and Latin America [52,53], while the risk of dengue transmission in the U.S. increases as the mosquito vector, *Ae. aegypti *broadens its range across the Mexico-US border [54,55]. Conventional vector control methods employed to reduce mosquito-borne disease transmission have been unable to prevent increased disease incidence, and additional measures such as the release of transgenic mosquitoes carrying genes designed to impact vector competence, have been proposed as auxiliary tools to control dengue transmission [56]. These strategies, referred to as population replacement, are based on the hypothesis that an increased frequency in a vector population of a gene that interferes with a pathogen will result in the reduction or elimination of transmission of that pathogen [57].

Anti-pathogen effector genes that block or kill viral pathogens, and promoters that target effector gene expression to key tissues where the virus and mosquito host interact are essential components of population replacement strategies to control dengue transmission. Dengue viral invasion of mosquito host tissues is ubiquitous, however important tissue barriers relevant for mosquito infection and subsequent transmission include the midgut and the salivary glands [58]. Expression of anti-pathogen genes should be limited to a specific sex, time and tissue/organ in the mosquito to achieve the maximum effect on the pathogen, while minimizing potential fitness load on the insect [56]. Various promoters have been identified and tested for their capacity to efficiently regulate reporter gene and effector molecule expression in specific mosquito tissues. The promoter of a carboxypeptidase gene has been characterized functionally [59] and was used to drive expression of an anti-dengue siRNA effector molecule in the midgut epithelium of transgenic mosquitoes, resulting in reduced virus prevalence and mean intensities of infection [60].

Recent work showed that salivary gland promoter-directed expression of an anti-dengue siRNA effector molecule also results in reduced virus prevalence and mean intensities of infection in transgenic mosquitoes [25]. However, effector molecule expression, mediated by the salivary gland-specific *30K b *promoter, was limited spatially to only the distal lateral lobes. Since dengue viruses infect and replicate in all lobes of the salivary gland [58], it is possible that enhanced virus suppression and subsequent disruption of dengue transmission may be achieved by expression of anti-viral effector molecules throughout the entire salivary gland. The identification of additional promoters whose products have this desired spatial expression profile in the salivary glands of adult *Ae. aegypti *females will enhance efforts to interfere with dengue virus transmission within the mosquito vector.

Our findings have implications to the design of multiple and/or combinatorial promoter-effector molecule constructs that would target dengue virus throughout the entire salivary gland of *Ae. aegypti *mosquitoes. We postulate that effector molecule expression in all lobes of the salivary glands can be achieved best with the use of two promoters, one each from the proximal-lateral and distal-lateral/medial groups. A combination of promoters, from the medial group and the all-lateral group also can be used. Quantitative RT-PCR results indicate that a promoter from each of these groups should permit constitutive expression of effector molecules, before and after a blood meal. However, it is worth mentioning the moderate decrease in transcript abundance following a blood meal observed for two genes in the medial group.

Global transcriptome analyses of mosquito salivary gland responses to blood-feeding have been performed [61,62] and changes in the transcriptome of mosquito cells, and tissues, including the salivary glands, during virus infection have also been reported [63,64]. Quantitative variations in transcript accumulation following blood meals or virus infection were detected for a small percentage of all genes expressed in mosquito salivary glands. Furthermore, modulation is moderate (less than 3 fold) for most of the regulated genes, an observation consistent with our quantitative RT-PCR data. Nevertheless, work to investigate modulation of gene expression in the salivary glands of dengue-infected mosquitoes is lacking and necessary.

## Conclusion

The work described here identifies five distinct groups of transcript localization patterns in the salivary glands of *Ae. aegypti *mosquitoes. Quantitative RT-PCR analysis indicates that seven selected genes are expressed constitutively and that salivary gland gene expression is not modulated significantly by blood-feeding except for genes in the medial group, which were observed to be down-regulated. Additionally, a morphologically-distinct cell type was identified in the anterior-most region of the proximal lateral lobe. Our findings emphasize the importance of investigating further the complex transcriptional and potentially translational regulation of gene expression in the salivary glands of mosquitoes. Genetically-modified mosquitoes expressing anti-dengue effector molecules exclusively in the distal lateral lobes resulted in decreased virus prevalence and mean intensities of infection. Dengue virus transmission potential of those transgenic mosquitoes was reduced strongly. Here we propose the use of at least two promoters to drive expression of anti-dengue molecules within the entire salivary gland to more efficiently reduce, or block disease transmission.

## Competing interests

Authors declare that they have no competing interests.

## Authors' contributions

JJ prepared hybridization *in situ *probes, performed hybridization *in situ *experiments, conducted quantitative real time RT-PCR experiments and analyses, participated in the design and coordination of the study and drafted the manuscript. UN-U and AM performed RT-PCR amplifications and cloning, prepared hybridization *in situ *probes and performed hybridization *in situ *experiments. BAMG carried out the transmission electron microscopy experiments. JC dissected salivary glands. PFPP interpreted transmission electron microscopy images and helped to draft the manuscript. WA and AAJ read and helped to draft the manuscript. OM conceived of the study, participated in its design and coordination and helped to draft the manuscript. All authors read and approved the final manuscript.

## Supplementary Material

Additional File 1 **Oligonucleotide primers for in situ probes and oligonucleotide primers for quantitative real-time RT-PCR**. Click here for file
