# Supplementary figures and images for: Spatial mapping of gene expression in the salivary glands of the dengue vector mosquito, aedes aegypti
Source: Parasit Vectors. 2011 Jan 4;4:1. doi: 10.1186/1756-3305-4-1 (PMC3043528; doi:10.1186/1756-3305-4-1)

## Slide 1
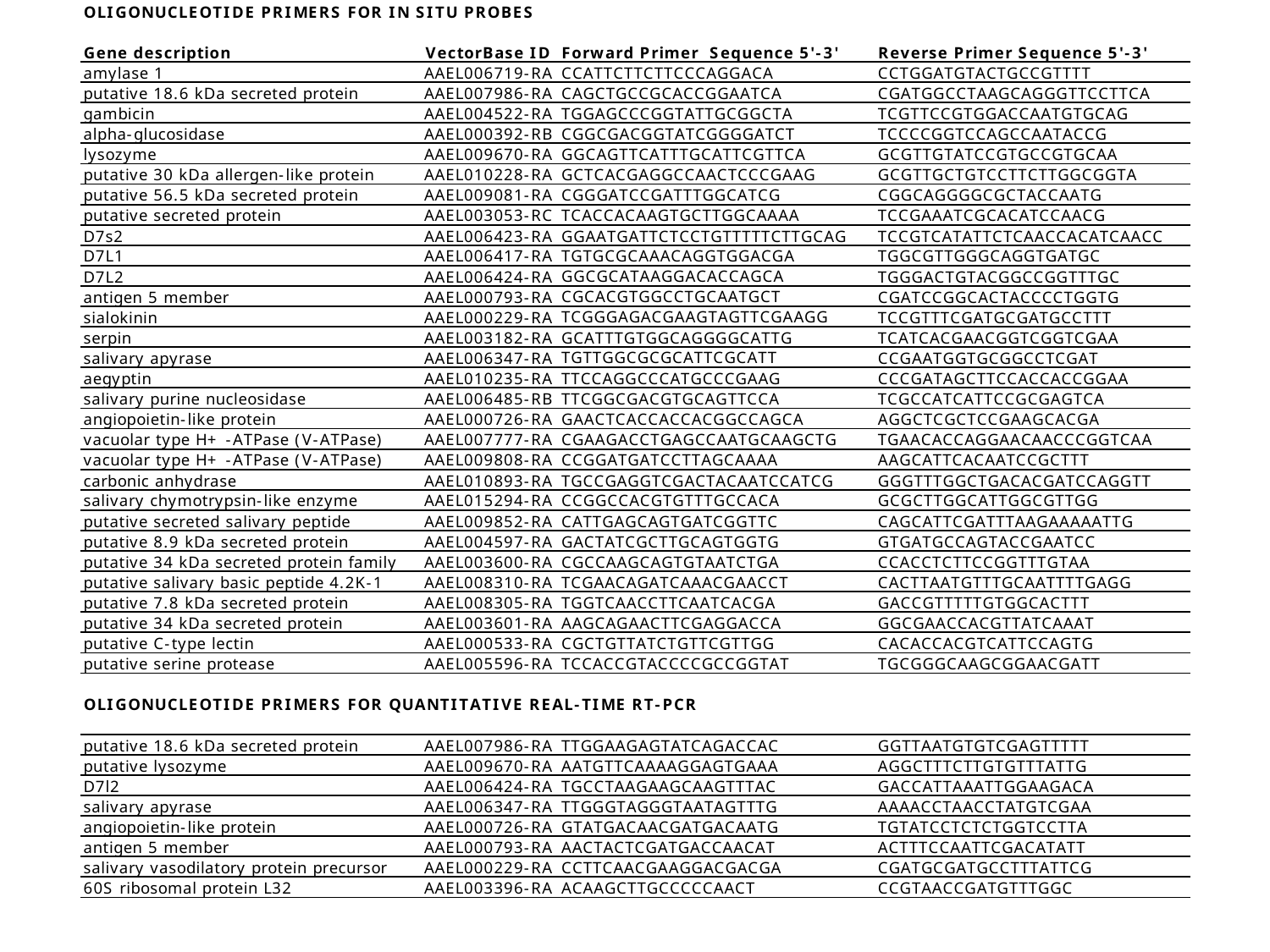

Supplement: Additional File 1 — Oligonucleotide primers for in situ probes and oligonucleotide primers for quantitative real-time RT-PCR. [file 1756-3305-4-1-S1.PPT]
